# From Integrated Care to Integrating Care: A Conceptual Framework of Behavioural Processes Underlying Effective Collaboration in Care

**DOI:** 10.5334/ijic.7446

**Published:** 2023-10-18

**Authors:** Karin Kee, Henk Nies, Marieke van Wieringen, Bianca Beersma

**Affiliations:** 1 Department of Organization Sciences, Vrije Universiteit Amsterdam, De Boelelaan 1105, 1081 HV Amsterdam, the Netherlands

**Keywords:** integrated care, collaboration, social motivation, epistemic motivation, voice behaviour

## Abstract

**Introduction:**

At all levels, effective collaboration between actors with different backgrounds lies at the heart of integrated care. Much attention has been given to the structural features underlying integrated care, but even under structurally similar circumstances, the effectiveness of collaboration varies largely.

**Theory and methods:**

Social and organizational psychological research shows that the extent to which collaboration is effective depends on actors’ behaviours. We leverage insights from these two research fields and build a conceptual framework that helps untangle the behavioural processes underlying effective collaboration.

**Results:**

We delineate that effective collaboration can be realized when actors (1) speak up about their interests, values, and perspectives (voice behaviour), (2) listen to the information that is shared by others, and (3) thoroughly process this information. We describe these behaviours and explain the motivations and conditions driving these. In doing so, we offer a conceptual framework that can be used to explain what makes actors collaborate effectively and how collaboration can be enhanced.

**Discussion and conclusion:**

Fostering effective collaboration takes time and adequate conditions, fitting the particular context. As this context continuously changes, the processes and conditions require continuous attention. Integrated care, therefore, actually requires a carefully designed process of *integrating* care.

## Introduction

Over the past decades, the urgency to provide holistic and seamless care for individuals, taking their specific care needs and preferences into consideration, has become stronger [1,2,3]. Yet, realizing such integrated care services is often easier said than done, as many impeding factors exist [4,5,6]. Studies to date have focused, in particular, on barriers to structural and functional integration [7,8]. The former refers to “physical, operational, financial, or legal ties among operating units within a system,” and the latter to “formal, written policies, and protocols for activities that coordinate and support accountability and decision-making among operating units” [9, p.201]. Scholars, in this regard, have found that distance between providers, differences in regulation, unclear roles and responsibilities, and a lack of joint funding hinder collaboration and, therewith, hamper care integration [6,8,10,11].

Yet, even under similar systems and conditions, considerable variation in the outcomes of integrated care projects has been found. For example, ostensibly successful pilot projects were found to not always transfer to other locations, despite the same structures and protocols having been implemented in these locations [12]. Similarly, an initiative in the Netherlands aimed at integrating care services for patients suffering from COPD/Asthma, Diabetes, or cardiovascular disease showed mixed results: from the 113 different care groups that participated in the initiative, some were able to improve care services in such a way that it enhanced care outcomes for clients, whereas other groups were unsuccessful in realizing this [13,14].

Several factors might explain such outcome variation and challenges with replicating successful care integration, such as, for example, regression to the mean and unobserved structural or contextual differences across locations. Besides these factors, scholars and practitioners focusing on care integration increasingly recognize that to realize integrated care services, it is important to focus on more than structures and systems. Instead, the “human dimension” of care integration needs to be taken into consideration as well [1,4,13,15]. After all, regardless of the type, level, and breadth of the integration initiative, all integrated care can be regarded as “people’s work”. That is, people (henceforth: actors) from different teams and organizations, with their own educational and professional backgrounds, values, and interests, as well as those of the team or organization they represent, need to collaborate in order to coordinate care around clients’ needs [16,17,18]. The inherent differentiation among actors that is central to integrated care initiatives is important: it gives collaboration its value, as the unique knowledge and perspectives actors can bring to the table allow them to generate unique, personalized solutions to the care needs of clients [15,19] and to attune to the local and national political and societal context. Yet, these differences between actors also make collaboration particularly challenging [16,18,19,20,21]. As the aforementioned mixed findings demonstrate, some groups of actors seem to be better able to collaborate effectively and actually utilize their differences in order to generate improved care outcomes, whereas other groups are less successful in doing so.

Over the last decade, scholars and practitioners in the field of integrated care have gradually begun to explore what is needed for actors to collaborate effectively [4,13,19,21,22,23]. While scholars have argued that effective joint working is a matter of ‘teamwork’ [24,25], a lack of clarity exists regarding what ‘effective teamwork’ entails in practice, as the behavioural processes underlying ‘effective teamwork’ have not been studied in the field of integrated care. Hence, *how* actors can collaborate in such a way that they are able to utilize the differences that exist between them in order to realize outcomes that benefit *all* actors involved in the collaborative effort as optimally as possible is still a pressing question in need of answers. Second and relatedly, a wide range of factors have been empirically identified that have been shown to foster or hamper collaboration and, therewith, care integration. For instance, (a lack of) joint funding, shared workspaces, and shared goals have been found to enhance (hinder) collaboration [6,7,22,26,27]. However, *why* these interventions have been successful, that is, which mediating processes explain the success of these interventions, remains unclear.

In the current paper, we contribute to the literature on integrated care by presenting a conceptual framework that elucidates *how* actors can collaborate effectively (i.e., which behaviours foster effective collaboration across different types of care integration efforts, that is, from small client-focused initiatives to larger, inter-organizational projects), *when* they do so (i.e., which factors/interventions foster effective collaboration), and *why* (i.e., why certain factors/interventions have the effects they have by uncovering what contributes to their effectiveness). To do so, we draw upon research from the fields of social and organizational psychology, in which scholars have extensively studied how effective collaboration between actors can be realized [28,29,30]. We leverage these insights and theorize that the extent to which collaboration, and consequently, care integration, is effective depends on the *behaviours* the involved actors demonstrate. That is, whether actors (1) speak up and share their interests, values, and perspectives with each other, (2) listen to the information that is shared by others, and (3) process this information so as to develop a thorough understanding of each other’s interests, values, and perspectives [28,29,30]. In what follows, we describe these behaviours and explain the motivations and conditions driving these.

## Integrated care as a joint decision-making process

The delivery of integrated care services can, at its very basis, be regarded as a *joint decision-making process* in which multiple actors with different backgrounds, interests, values, perspectives, and expertise try to make joint decisions regarding the delivery of integrated care services [cf. 31,32,33]. Such decision-making processes occur at different levels [4]. For example, care professionals, clients, and informal carers need to coordinate care services around individual clients and, thus, need to make decisions, but so do policymakers of local and national governments and funding agencies (e.g. healthcare insurers) and executives of care providers.

Regardless of the level of the integration initiative, arguably, more optimal joint decisions about care services can be made when actors coordinate their actions and jointly decide what is needed for certain clients and who is able to provide this, and when. Consider, for instance, a situation in which caregivers give in to the needs and wishes of a client for additional care. While this may benefit the client, it may hamper the caregivers, whose workload further increases. In contrast, when actors collaborate and collectively design a solution to a client’s care needs that considers actors’ underlying interests and perspectives, this could result in a more optimal outcome for *all* actors involved [34]. The latter has been referred to as a ‘win-win’ outcome by Goodwin [35, p.257], as all actors benefit.

Yet, realizing joint outcomes that benefit all actors isn’t always easy. Differences in backgrounds, interests, values, perspectives, and expertise, for instance, can result in misunderstandings and can hamper the decision-making process between actors [15,21]. Additionally, actors may face mixed motives: they may have reasons to collaborate but also have reasons to focus mainly on their own interests [20]. Actors usually intend to deliver high-quality care and need each other to reach this goal, which is the reason to collaborate [36]. Simultaneously, actors cannot neglect their own agendas. For instance, healthcare insurers aim to operate on a sound financial basis and, thus, may place limits on the funding of care. Similarly, care organizations all need enough qualified personnel and may compete with other organizations for human capital. Thus, when making decisions, the involved actors may work with others to pursue shared goals but, at the same time, are unlikely to lose sight of their own interests [20,35]. What further adds to this complexity is that the benefits associated with integrated care as well as the effort required to make integration happen, are usually not evenly distributed among actors [35,37]. Resultantly, actors may perceive that some ‘win’, while others lose from collaborating, or, at the very least, that some have more to gain or lose than others. Hence, it can be challenging for actors to perceive problems as jointly faced and requiring collaborative action [38].

### The development of a conceptual framework

How, then, can actors collaborate effectively despite the challenging conditions they face and what can be done to enhance collaboration? To advance the literature and help practitioners, we aimed to develop a conceptual framework to answer this question. We searched for previous theorizing that possessed three characteristics. First, the theory should delineate *how* groups of actors can collaborate in a way that they are able to utilize the differences that exist between them in order to realize outcomes that benefit *all* actors involved in the collaborative effort. The latter is particularly important when integrating care services and is, in fact, the central idea behind integrated care [1,35,39]. Second, the theory should *describe under what conditions* such effective collaboration could be realized. Third, and finally, the theory should be generic and, hence, should shine a light on how diverse groups of actors at a variety of levels and hierarchical positions could collaborate effectively.

Following our conceptualization of integrated care as a *joint decision-making process*, we consulted the literature on this topic, which drew us to social and organizational psychological research. Studies on joint decision-making in these two research fields show that it is not ubiquitous for actors to realize high-quality outcomes that meet all actors’ interests and needs [40,41,42]. Specifically, studies show that both diversity among actors and the mixed-motive nature of many (group) decision-making tasks can hamper effective decision-making in practice [28,29]. In other words, the challenges actors involved in the process of integrating care services face are not unique: they are inherent to joint decision-making, regardless of the specific topic the integration efforts pertain to.

Insights obtained by social and organizational psychologists, who have extensively studied the intricacies of group decision-making processes, could shed new light on these challenges of integrating care. Throughout the 1980s and 1990s, numerous studies were carried out on group decision-making, which focused primarily on either cognitive or motivational aspects of joint decision-making. Cognition refers to mental processes involved in acquiring, processing, and utilizing information, which are essential steps in decision-making processes as they enhance understanding, support problem-solving, and enable actors to make informed choices [28]. Actors can differ in the amount of information they are willing to acquire, process, and utilize, as, for instance, becomes apparent in a study by Stasser and Titus [41]. The scholars observed that actors sometimes fell short in utilizing the unique information all actors held and, consequently, failed to find a solution that best fit all actors’ underlying interests. Motivation, in turn, refers to the effort individuals are willing to exert and their level of engagement. Studies on groupthink and defective decision-making, for instance, found that actors may be motivated to self-censor and self-enhance so as to secure favorable outcomes for themselves [43]. While a comprehensive examination of all theories on cognitive and motivational aspects of group decision-making is beyond the scope of this manuscript, De Dreu et al. [28] provide a comprehensive analysis and synthesis of the existing literature; interested readers are encouraged to consult their work for a more thorough exploration of the topic.

Around the turn of the century, scholars started to realize that the complexities of group decision-making could not be fully explained by the cognitive approach alone, as it lacked a comprehensive understanding of human motivation – and vice versa [28,29,42,44]. Instead, scholars discovered that cognition and motivation interact [45,46]. For instance, actors’ motivation influences the amount of effort actors are willing to put into acquiring, processing, and utilizing information. Thus, it is important to look at the joint influence of cognition and motivation, as these will determine the behaviours actors will demonstrate during decision-making processes, which, in turn, will influence the types of outcomes actors will reach [28,29]. In 2008, De Dreu, Nijstad, and Van Knippenberg [28] bundled these perspectives (and, hence, theories that up until that point had been considered in isolation) into a comprehensive, overarching framework that can be used for understanding the dynamics of joint decision-making, the “Motivated Information Processing in Groups-Model” (MIP-G).

Besides integrating a wide variety of theories, the MIP-G model is renowned for shedding light on the factors that contribute to effective joint decision-making in groups and its applicability across various contexts. These include cross-functional teams that perform non-routine and complex tasks [47], army cadets working on a dynamic, ambiguous decision-making task [48], and top management teams agreeing upon innovations proposed by team members [49]. What the aforementioned studies have in common is that actors have different perspectives and interests that must be integrated in order to come to a workable solution for all actors involved. The same is true for integrated care initiatives, which bring together various actors with different backgrounds, interests, values, perspectives, and expertise to integrate care services around clients’ needs and aspirations [4,15,21,50]. Hence, it can be assumed that the MIP-G model offers valuable insights for the field of integrated care.

MIP-G presumes that effective collaboration can be achieved when actors 1) carefully listen to each other and 2) thoroughly process the information that is shared by others in order to develop a thorough understanding of each other’s interests, values, and perspectives. Yet, before actors can demonstrate the two aforementioned behaviours, they should, first of all, *share* their interests, values, and perspectives with the other actors involved [15,30]. Doing so is crucial, as only through the explicit voicing of interests and ideas can actors discover ways in which mutual benefit(s) can be realized [21,35]. Valentijn et al. [39] showed, in this regard, that the success of different primary care programmes to support the management of chronic diseases was not related to shared ambition but, instead, dependent on the extent to which actors *voiced* their interests. In groups where actors remained silent about their interests and failed to share their ideas and perspectives, the achievement of collective outcomes was hindered. Conversely, groups in which actors voiced their interests and perspectives were able to successfully attain joint outcomes. This is in line with work by Allen et al. [51], which shows that by expressing their views, actors were able to collaboratively make decisions regarding the discharge of patients from the hospital to home care services that satisfied all involved actors. Hence, *voice* is crucial for actors to realize joint outcomes [21,35,39].

MIP-G theory implicitly assumes that actors always share their perspectives and ideas, making communication not a separate step in the model. Yet, both Goodwin [35] and Valentijn et al. [39] point out that this may not always be the case, with possible detrimental consequences. Following the emphasis they place on *voice*, we consulted the literature on *voice behaviour* – a field of study concerned with the question of when and how actors become willing to speak up [30]. Studies on voice behaviour have shown that speaking up and sharing one’s interests, ideas, and perspectives is not always evident, especially in healthcare settings, for instance, because of status differences between actors [52]. This, in turn, may hamper joint decision-making [53]. Therefore, we develop a conceptual framework that describes that not two but *three* behaviours are conducive to joint decision-making and, as such, to collaboration. Specifically, we argue that in order to reach the ideal of patient-centred integrated care services, actors first and foremost need to engage in a carefully designed process of *integrating care* – the latter is a verb, referring to the three behaviours actors should demonstrate. In what follows, we discuss the three behaviours in more detail.

## Behaviours, motivations, and perceptions in effective collaboration

### Speaking up and sharing one’s interests, values, and perspectives: voice behaviour

When integrating care services, it is important that actors are aware of each other’s interests, values, and perspectives [35,39]. Hence, it is crucial that actors engage in “voice behaviour”. Voice behaviour can be defined as the voluntary communication of perspectives, ideas, and concerns, through which actors attempt to have a say in and potentially influence matters that affect their work or lives [30].

*Illustration 1*

The importance of voice behaviour for care integration becomes apparent in a study on integrated transitional care in Australia [51]. Study findings indicated that healthcare practitioners used a range of communication processes, such as multidisciplinary team discussions, to optimize smooth transitions from hospital care to home care for patients. During multidisciplinary team discussions, care professionals engaged in a *decision-making process* and decided whether a patient was ready to be discharged from the hospital. By speaking up, allied health professionals were able to prevent premature patient discharge to home care.

*“The doctors would see something completely different from what we, allied health, would see. Allied health would be like: ‘They’ve come in three times in the past.’ And the medical team: ‘Well, their white cell markers are fine, so we’re happy for them to go.’ And then we’re like: ‘No!!’”* [51, p.5].

Many studies show that voice behaviour is not ubiquitous, especially in healthcare settings [52]. The well-entrenched hierarchy that characterizes healthcare settings can make speaking up particularly difficult for certain actors. Out of fear of receiving hurtful comments or negative appraisals, in particular, actors lower in (self-perceived) hierarchy and status are generally hesitant to speak up [53]. Wu et al. [52], for instance, described that the dismissive and authoritative attitude some physicians adopted in their interaction with nurses, significantly lowered the willingness of nurses to speak up. Yet, silence on the side of one of the actors involved in integrating care can lead to suboptimal decisions being made, as not all perspectives, interests, values, and ideas become visible.

As voice behaviour is crucial for bringing about effective collaboration, it is important to know how actors can be encouraged to demonstrate this type of behaviour. Research has shown that two factors, in particular, influence actors’ willingness to engage in voice behaviour [30]. First, voice is influenced by actors’ efficacy beliefs. That is, if actors believe they will be listened to and others will act upon their voice, they become more willing to speak up. In contrast, when actors perceive nothing is done with their input, they are more inclined to remain silent. Second, voice behaviour is influenced by actors’ safety perceptions [54]. Whereas actors, through exhibiting voice behaviour can share valuable ideas and perspectives, this is not to say that others always respond positively to this input. In fact, research has shown that even actors who sincerely wish to be open to the ideas and concerns of others may feel threatened by this input or may not always recognize the importance of this input and therefore respond in a defensive manner [55]. Out of fear of jeopardizing their relationship with others, particularly lower status actors may become reluctant to speak up [52,53]. Care recipients, for instance, may believe that their physicians perceive them to be “bad patients” or “troublemakers”, once they exhibit voice behaviour [56]. In contrast, when actors consider it safe to speak up, that is, when they believe there are no negative consequences associated with this behaviour, their willingness to make their voices heard increases. This is, for instance, the case when higher-status actors solicit input and engage in consultative behaviour, because, these behaviours signal receptivity to voice behaviour [57]. Thus, although engaging in voice behaviour may seem self-evident, in practice, two essential conditions must be met before actors become inclined to demonstrate this behaviour.

### Listening to the information that is shared by other actors

Whereas an important first step in the process of integrating care is that actors speak up, it is just as important that they pay attention and listen to the information that others share. Whether actors listen to and consider the interests, values, and perspectives of others depends on their level of social motivation [28]. Social motivation refers to how actors weigh their own and others’ interests in decision-making [30]. Actors can have a so called ‘proself motivation’ and primarily strive to maximize their own outcomes. Consequently, these actors tend to have no or less regard for the interests, values, and objectives of others [58] and may even ignore the information that is shared by them [28]. In contrast, actors can also have a so called ‘prosocial motivation’, which means that they are concerned with the interests, values, and perspectives of other actors as well as with their own [59]. As actors with a prosocial motive aim to reach joint decisions that value and incorporate both their own and other actors’ interests and ideas, they are more likely to listen to the unique information that is shared by others [40].

Actors with a proself motivation, who focus on their own interests, may arguably be unlikely to participate in integrated care endeavours in the first place [60], unless these endeavours enable them to pursue their personal goals. Actors may not always be completely prosocially motivated either and, thus, willing to listen to the input that is shared by others. For instance, higher-status actors may (falsely) assume that they do not need lower-status actors’ input and, therefore, may ignore this [52].

*Illustration 2*

The detrimental consequences of a lack of listening behaviour became apparent in a cross-European study on informal carers’ roles and needs in integration initiatives [61]. Interviews with 48 informal carers revealed that many of them perceived that care professionals were mainly focused on the needs and wishes of the client. Information that was shared by the informal carers regarding their own personal situation and, as such, their (in)ability to provide informal care was not always thoroughly listened to.

*Informal carer: “I don’t think that they understand our situation. I am in much poorer health than what they believe. (…) I have had two big heart attacks and two big operations. And my back is not in good shape.”*

Ultimately, informal carers felt that professionals may have assumed that they would serve as informal caregivers by default, whereas this was not always feasible [61, p.4].

### Thoroughly processing the information that is shared by others

When actors speak up about their interests, values, and perspectives and listen to the input that is shared by others, a lot of new information becomes available to them. In order to successfully leverage this information for care integration, actors need to accurately *process the information*. Whereas listening to the ideas and perspectives voiced by other actors requires *being open to* and *collecting* new input and perspectives, *thorough information processing* requires actors to think deeply about the information that is shared and consider how it can be used to achieve the desired joint outcome(s) [62].

Whether actors are willing to thoroughly process the information that is shared with them, and, as such, expend effort to achieve an accurate understanding of other actors’ interests, values, and perspectives, depends on their level of so-called epistemic motivation [28,29]. Actors with low levels of epistemic motivation tend to process information in a heuristic manner. This means that they show little effort to process new information that is brought up by other actors and, instead, make judgments through quick and effortless processing of information, largely based on prior knowledge, “standard” solutions, and rules of thumb [28,43]. In contrast, actors with higher levels of epistemic motivation tend to systematically process information and, as such, suspend judgment and engage in a more effortful and deliberate processing of information to come to a decision [64].

When integrating care, actors benefit from higher levels of epistemic motivation. After all, the process of integrating care tends to be characterized by high complexity due to the differences between actors, the complexity of the context, as well as the mixed-motive nature of integrated care [1,20]. Under higher levels of epistemic motivation, actors are able to develop a thorough understanding of the interests, values, and perspectives of other actors. This allows them to search for and realize integrative potential to a greater extent than when actors expend little effort to process the information that is shared with them [28].

*Illustration 3*

A study on an integrated team approach to fall prevention for older home care clients in Canada illustrates the importance of the thorough processing of new information by actors [31]. The team members involved (e.g., registered nurse, physiotherapist, occupational therapist) all utilized their own standardized clinical assessment tools but also thoroughly listened to and *processed the insights* that were shared with them by the other members during team meetings. By doing so, the care professionals were able to utilize the unique types of information they all brought to the table, which allowed them to develop personalized care plans that fit the needs, goals, and values of individual clients.

*“What I’m finding at the group meetings is how I don’t seem to get the same kind of information as some other people. That there are components that if we hadn’t had all these people going in to get information, we might not have gotten as complete a picture as we get when we’re in a group.”*

*“People share different things, and so it gave more for each of us to think about and a better-rounded view of the client as well.”* [31, p.7]

## Person-based and situation-based antecedents as enhancers of collaboration

In the previous section, we have discussed three types of behaviours that social and organizational psychological research has shown to be conducive to collaboration. We have also delineated that whether actors demonstrate these behaviours in practice is dependent on their perceptions and motivational states. Actors’ perceptions and motivational states, in turn, are – according to MIP-G and voice theory – influenced by two sets of antecedents: person-based and situation-based antecedents.

Person-based antecedents refer to actors’ personality traits. Some actors possess personality traits that naturally make them more willing to speak up or which increase their epistemic or prosocial motivation. For instance, actors with personality traits such as extraversion and assertiveness are less concerned with the possible negative outcomes of voice behaviour, and, therefore, naturally more inclined to speak up compared to actors without such a trait [65] (see for overviews of person-based antecedents De Dreu et al., 2008 [28] and Morrison, 2014 [30]).

Because personality traits affect the perceptions and motivation of actors and, thereby, the behaviour they are likely to demonstrate, the extent to which actors will collaborate effectively is, to a certain extent, dependent on actors’ personality traits. For instance, if all team members have a high inclination to speak up, the group has a higher chance of reaching a creative joint decision based on the input of all members involved than a team that consists of actors who are more inclined to remain silent [66]. Similarly, a team that consists of members who all value cooperativeness is more likely to reach joint decisions that are beneficial to all actors involved than a team that consists of actors who go for their own interests [67]. Thus, the first way in which care integration can be enhanced is by forming teams of actors who possess those personality traits that are conducive to collaboration (although this is often easier said than done, see below for a discussion).

Situation-based antecedents, in turn, refer to the context in which actors operate or the structure under which their collaboration takes place [28,68]. Just like person-based antecedents, they influence actors’ perceptions and motivational states. For instance, research has shown that when actors must make decisions under time pressure (a situational factor), they perceive it is less effective to engage in voice behaviour, as they believe there is little room for a (possible) dissenting opinion [69].

As situation-based antecedents can affect actors’ perceptions and motivation, changing the context of the decision-making process, sometimes even in a subtle way, can alter actors’ perceptions and motivation, and, thereby, their behaviour. For instance, reducing time pressure increases actors’ willingness to engage in voice behaviour [30]. Thus, a second way in which the integration of care services can be enhanced is by creating a context that is favourable for collaboration.

All in all, person-based and situational antecedents affect the extent to which effective collaboration can be realized [28]. Prior research, however, suggests that the latter tends to be more influential than the former [60,68]. This is an important insight, as it is often more feasible to change the situational conditions than it is to alter group composition. After all, changing the team composition in integrated care initiatives or in policy-making contexts is not always feasible. Therefore, the next question to be addressed is which situation-based antecedents influence actors’ perceptions and motivational states, and according to what principles the context can be altered to enhance care integration.

### Situation-based antecedents that influence actors’ voice safety and efficacy perceptions

As discussed previously, if actors perceive that it is safe to speak up and believe that their voice will be listened to and acted upon, their inclination to speak up increases [30,52]. These perceptions are shaped by a variety of factors. First, the most influential factor affecting actors’ voice perceptions is the safety of the relationship actors have with others [53; 55]. Actors consider it to be safer to speak up when they trust the other actors and feel recognized by them [26; 70]. For instance, scholars have found that a shadowing exercise, in which actors spent a morning or afternoon at each other’s workplaces to become familiar with one another and their work was an effective tool in building trust and cooperation, which, in turn, enhanced actors’ safety perceptions [53].

Second, soliciting feedback and emphasizing the unique knowledge actors can bring to the table, may enhance voice behaviour as well [57]. Nembhard and Edmondson [71], for instance, have shown that doctors who emphasized the unique knowledge and expertise nurses brought to an integration project, ensured that nurses felt more at ease with speaking up, as they believed their input was welcome, and, therefore, effective. As such, when (higher-status) actors engage in consultative behaviours and articulate the importance of other actors’ voice behaviour, they signal a receptivity to voice behaviour, making it more likely that other (lower-status) actors speak up.

Third, power cues play a role. Collaborative endeavours between actors may be full of (implicit) power imbalances, such as a manager sitting at the head of the table [72]. Such power imbalances could make particular actors uncomfortable and may lower their efficacy and safety perceptions. Kee et al. [53], for instance, have shown how the use of policy jargon during meetings with managers and policymakers hindered the voice behaviour of nurses, as the latter could not follow along and, therefore, could not share their ideas and suggestions. In contrast, the use of layman’s language could make actors feel more confident [17]. Attire also contains power cues. That is, the manager’s tie and the doctor’s white coat are associated with a certain level of authority and power [73]. When worn during meetings with patients and their relatives, a power imbalance may arise, which could make patients and their relatives hesitant to speak up. By softening the power cues, actors may become more willing to share their input.

### Situation-based antecedents that influence social motivation

Situation-based antecedents can also induce a prosocial motivation in actors, making it more likely that actors listen to one another [28]. First, the reward structures under which collaboration takes place influence social motivation. Although scholars and practitioners in the field of integrated care increasingly acknowledge the “human dimension” of care integration [1,4,15,21], having the right reward structure in place remains an important requirement for collaborative efforts, precisely because reward structures have been shown to influence social motivation, with individual incentives promoting proself, and collective incentives promoting prosocial motivation [28]. This implies that even if managers emphasize collaboration, this is unlikely to have a strong effect if the reward structure in their organization continues to incentivize individual interests, values, and outcomes [60]. It is therefore important to develop a reward structure that is in line with the joint goal that is being pursued.

Second, actors’ social motivation depends on how the collaborative endeavour is framed [29]. Actors are more likely to adopt a proself motivation when differences between them are emphasized without reference to the added value of these differences for collaboration. This may be the case when actors use subtle framings such as “us and them” (for instance, when talking about differences in vision or goals) [27]. In contrast, when the other actors are referred to as “partners” or when a discourse that centres around “we” is used, the shared ownership of the integrated care initiative is emphasized, and this makes actors more likely to adopt a prosocial motivation [40].

Third, location matters. Dickinson and Joos [74] have shown how the layout of a hospital complex affected collaboration. Specifically, they have shown that the physical separation between actors, as they were based in different buildings, contributed to an atmosphere that was observed to be strained and consisted of stark inter-professional borders. In contrast, the use of integrated settings, such as co-located spaces, has been shown to increase the proximity between actors [75], allowing them to develop a prosocial motivation.

Finally, actors are more likely to adopt a prosocial motivation when they aim for long-term collaboration or when possible future interactions between the various actors involved are emphasized [28,29]. A shared, long-term vision in which actors clearly articulate the future stake they may have in common in the integrated care initiative is helpful in creating conducive circumstances for collaboration [22,35,39].

### Situation-based antecedents that influence epistemic motivation

A number of antecedents induce high levels of epistemic motivation in actors. First, the available time actors have to collaborate and make decisions about the delivery of care services to individual clients matters. Time pressure lowers actors’ epistemic motivation, because under time pressure, the speed of decision-making becomes more important than information processing and decision accuracy [29]. In contrast, when actors have enough time to decide, they are more likely to undertake additional efforts to develop a thorough understanding of each other’s perspectives and interests [63].

Second, epistemic motivation increases when critical thinking is encouraged among actors [76]. When actors are stimulated to engage in critical thinking, they may come to realize that other actors hold divergent preferences, values, ideas, or opinions. These divergent perspectives challenge actors’ confidence in their own preferences, ideas, or opinions, which encourages a search for and processing of new and additional information. This also underlines the importance of voice behaviour, as through the practice of speaking up, divergent ideas and perspectives may become visible in a safe context.

Finally, the physical setting in which the meeting takes place matters. That is, epistemic motivation is reduced when there is a high level of ambient noise, for instance, when a meeting takes place in a noisy room or when meetings are disturbed by background noises [77], such as beepers and phone calls. Moreover, when meetings become too lengthy and individuals become fatigued, the latter may also have lower levels of epistemic motivation [28].

## Integrating care: An overview of aspects that enhance the process

**Figure 1:**
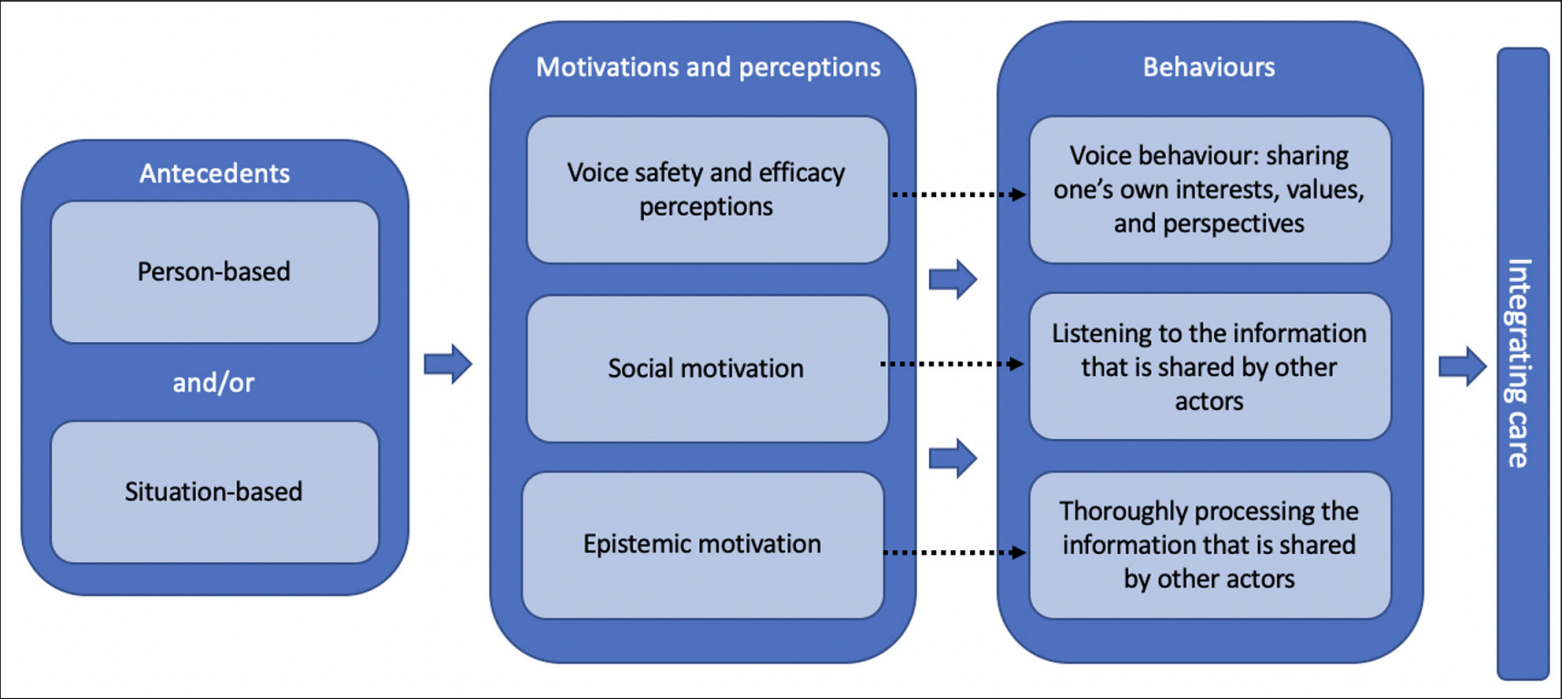
Conceptual overview of the process of integrating care.

One of the most pressing challenges scholars and practitioners in the field of integrated care currently face is to delineate what makes actors collaborate effectively [15; 23]. By drawing upon insights from the fields of social and organizational psychology, we have built a conceptual framework that attends to what collaboration entails in terms of the behaviour of actors involved. The framework is illustrated in Figure 1.

Specifically, we have argued that for actors to realize *joint outcomes* and, thus, to collaborate effectively, it is necessary that actors (1) exhibit voice behaviour and share their interests, values, and perspectives, (2) listen to what is shared by other actors, and (3) thoroughly process this information in order to develop a thorough understanding of each other’s interests, values, and perspectives.

Whether actors demonstrate these three types of behaviour, in turn, depends on actors’ motivation and the perceptions they hold. Specifically, whether actors make their voices heard depends on if they consider it safe and effective to do so. Whether actors are open to each other’s perspectives and, as such, willing to listen to one another depends on their levels of social motivation. Finally, actors’ information processing depends on their level of epistemic motivation. Whereas the three behavioural states, and the accompanying motivational states and perceptions, were discussed separately, all three are essential for integrating care. If actors, for example, are willing to share their ideas but do not thoroughly process the information that is shared, it is likely that the integration attempt will remain unsuccessful.

Actors’ perceptions and motivational states are, in turn, influenced by person-based and situation-based antecedents. Some actors possess personality traits (person-based antecedents) that naturally make them more inclined to demonstrate the three types of behaviour outlined above. Hence, these actors could be asked to participate in the integration project. Further, actors could be encouraged to demonstrate the three types of behaviour that are conducive to collaboration as well by (slightly) altering the context of the collaborative endeavour.

### Focal points when integrating care services

While our conceptual framework describes how the delivery of integrated care services depends on the behaviour actors demonstrate, this is not to say that care integration is a matter of “behaviour” alone. In fact, by making the motivations and perspectives underlying behavior in the process of integrating care, as well as the person- and situation-based antecedents of these, explicit (see Figure 1), our framework shows that it remains just as important for actors, such as leaders, to tackle well-known barriers to integrated care. Limited time and resources and a lack of shared goals [6,74], among others, may hamper actors’ ability to demonstrate the three behaviours that have been shown to be conducive to collaboration. For instance, time constraints may hinder actors’ ability to exhibit voice behaviour, and to listen to and process information shared by others. Similarly, a lack of funding incentives to support collaborative work and a lack of joint working space may prevent actors from becoming prosocially motivated. It is, therefore, important to search for barriers in the design of the collaborative process and attempt to minimize these.

Relatedly, our framework implies that there is a need to align the various situation-based antecedents to enhance collaboration. For instance, if actors consequently speak of “we” and discuss joint goals together (which enhances prosocial motivation) but budgets are distributed between them in a competitive manner (which enhances proself motivation) and actors have to deal with time constraints (which lowers epistemic motivation), this is likely to not result in the desired effective collaboration. Therefore, it is important to search for inconsistencies in the context in which care integration takes place and to try to overcome these.

## Discussion

In this paper, our aim was to develop a better understanding of how effective collaboration between actors involved in care integration can be realized. By leveraging insights from social and organizational psychological research, we contribute to the literature on integrated care by offering a conceptual framework on the behavioural aspects of care integration. By doing so, we increase understanding of *how* actors can collaborate effectively and *when* they do so. Additionally, our framework sheds light on *why* well-known facilitators and barriers to collaboration have the effects they have by untangling the underlying behavioural mechanisms that explain the effects of such interventions. In what follows, we discuss our work in light of the existing literature.

From the notion that care integration can, in essence, be regarded as “people’s work”, we have theorized that the behaviours actors demonstrate will affect the extent to which they are able to collaborate effectively. Specifically, we have shown that effective collaboration is dependent on the extent to which actors voice their perspectives and ideas, attentively listen to, and thoroughly process the unique input shared by other actors. By doing so, we offer an explanation for why ostensibly successful pilot projects were found to not always transfer to other contexts [12,13]. Further, it explains why similar systems and structures do not lead to similar outcomes and why across systems and countries, similar barriers to integration exist. It is the behaviour of the actors that largely cause variations in outcomes, but, situation-based conditions also matter because they lead actors to engage in certain behaviours.

In extension, we have provided insight into how collaboration can be enhanced. For long, scholars and practitioners in the field of integrated care have assumed that effective collaboration is a matter of ‘having the right people in the right place’ [78,79]. Indeed, as we have theorized, some actors may naturally be inclined to demonstrate behaviours that are conducive to collaboration. Yet, even when actors involved in the process of integrating care do not possess the personality traits that are deemed favourable for this process, effective collaboration may still be realized by (slightly) altering the context of the collaborative endeavour. To this end, our framework can be used to *understand* (retrospectively) and to *predict* (prospectively) under what circumstances effectively collaboration can be realized. In recent years, scholars in the field of integrated care have reported under what circumstances collaboration turned out to be effective. For instance, the formulation of shared goals among actors [22,27], leaders who emphasize collaboration [80], joint working spaces [74], and joint funding [6,7,26] have been identified as enablers of collaboration. Our conceptual framework increases understanding of *why* precisely collaboration was enhanced under these circumstances. That is, these circumstances enabled actors to demonstrate the three types of behaviour that underlie effective collaboration.

In addition, our framework sheds light on what “good leadership” in integrated care may entail. Whereas studies identified “a lack of leadership” as a barrier to integration and “good leadership” as an enabler [6,7,80], little is known about what a “good leader” exactly does [35,81]. Zooming in on actors’ behaviour, our framework underscores the need for leaders to facilitate actors to engage in voice behaviour, listening behaviour, and information processing. While leaders may select those team members who are naturally inclined to demonstrate these types of behaviours (the person-based antecedents in our conceptual framework), a more feasible action is often to put effort into developing a context that is favourable for collaboration. The situation-based antecedents in our conceptual framework provide guidance on how to do so. Our framework does not only delineate how a fruitful context for effective collaboration can be developed, but also shows the other, more negative side, of the coin. That is, situation-based antecedents may also negatively affect actors’ motivation and perceptions [28,29]. For instance, if actors are stimulated by their leader to solely consider the goals of their own organization, this decreases the chance that they will have a prosocial motivation. What this essentially means is that when starting an initiative to integrate care, leaders need to carefully consider the people, the structure, and the context in which the collaborative endeavour takes place.

Leaders also play an important role in creating a level playing ground for all actors involved in the integration initiative. While all actors involved can contribute in their own unique ways, this is not to say that all actors are necessarily “equivalent.” That is, (perceived) differences in power and status may exist, which may hinder joint working [33,82,83]. Dos Santos and Giovanella [84], for instance, described how political stakeholders offered health administrators little room to speak up, causing them to have little influence on the decisions about new health policies in Brazil. Relatedly, research has shown that actors with relatively high status within the group tend to disregard the information shared by low-status actors [57,71]. Leaders can play a role here by inviting all actors for input, thereby enhancing actors’ voice efficacy beliefs. Similarly, leaders may induce a criticality norm in the group, causing actors to become more likely to listen to and process information shared by others [76].

A next step forward would be to test and further explore the proposed relationships in our conceptual framework in the context of integrated care settings. For instance, as a starting point, scholars could test (e.g., by means of survey research) whether the three behaviours outlined in our framework have a positive, significant effect on the extent to which actors involved in integrating care perceive the decision-making process and outcome as successful. Comparing results across different integrated care settings (ideally at different levels) would enable conclusions as to whether the framework is indeed generic, as proposed, or whether specific contextual moderators affect the influence of the three central behaviours on the success of care integration. Furthermore, it would be worthwhile to examine the interplay between different situation-based antecedents and to investigate if some antecedents are more influential than others.

## Conclusion

To reach integrated care, actors need to collaborate. Having the right people, structures, systems and processes in place, is one thing. Engaging in the most appropriate behaviours to achieve the desired collaboration is another. By drawing on literature from the fields of social and organizational psychology, we aimed to address this matter. In line with the notion that the integration of care services is in essence “people’s work”, we have demonstrated that the outcomes of collaborative endeavours depend on how actors interact. Specifically, we have argued that three types of behaviour enhance collaboration and have delineated how person-based and situational antecedents can influence these behaviours. As such the structures under which collaboration takes place configure behaviours of actors and how they demarcate their boundaries (i.e., responsibilities and ownership).

Our conceptual framework implies that the outcomes of integrating efforts are, to quite some extent, people- and context-dependent. Integrated care simply is not a predefined outcome exactly mirroring other pilots and examples. Integrated care is not a well-defined end-state. Rather, it is an ongoing process of adjusting, learning, improving, and being resilient in a context in which people, structures, systems, and societies are continuously changing and moving forward and backward. This underlines our argument that integrated care essentially revolves around *integrating care*. The latter is a verb, which refers to behaviour that requires ongoing attention.
